# Assessment of household settled dust via silicon nanomembrane analysis pipeline (SNAP)

**DOI:** 10.1016/j.eti.2025.104106

**Published:** 2025-02-26

**Authors:** Samantha S. Romanick, Gregory Madejski, Garrett Cashion, Andrew J. Berger, Alison Elder, James McGrath

**Affiliations:** a University of Rochester, Department of Biomedical Engineering, 480 Intercampus Dr, Rochester, NY 14627, United States; b University of Rochester, UR Nano, Department of Physics, 480 Intercampus Dr, Rochester, NY 14627, United States; c University of Rochester, Department of Environmental Medicine, 601 Elmwood Ave., Box 850, Rochester, NY 14642, United States; d University of Rochester, The Institute of Optics, Wilmot 121, Rochester, NY 14627, United States

**Keywords:** Particle capture, Microplastics, Microfibers, Raman spectroscopy, SEM

## Abstract

Humans spend 70–90 % of their time indoors. However, there is a significant lack of knowledge regarding human exposure to microplastic particles and fibers (MPs) within the indoor environment. Fibers comprise more than 90 % of household settled dust worldwide and have been found in indoor air. Studies have identified MPs larger than 50 μm in indoor dust, but little information is available regarding smaller airborne or settled particles. We have developed methods to detect plastic particles that are larger than 10 μm in settled household dust by distinguishing plastic from cellulosic, proteinaceous, and inorganic particles using Nile Red to stain for plastics and Trypan Blue to stain for cellulosic materials while proteinaceous and inorganic materials remain unstained but visible via transmission light microscopy on ultrathin silicon nitride nanomembranes. This method, which we term the Silicon Nanomembrane Analysis Pipeline (SNAP), allows household settled dust and other sample types to be collected and analyzed on nanomembranes by multiple modes of metrology *in situ*, avoiding the need to transfer particles to different substrates for capture vs. analysis. Specifically, particles analyzed by fluorescence staining and optical imaging was followed by polymer identification via Raman spectroscopy and subsequently characterized via scanning electron microscopy for size and surface morphology. Using this innovative approach, microplastic particles larger than 10 μm in diameter have been identified in all settled dust samples.

## 1. Introduction

Plastic pollution has devastating effects on the environment and wildlife, but the impact on human health is still unclear. Microplastics (MPs), a term defined in 2004 by Thompson et al., are small plastic particles measuring less than five millimeters in size that result from the environmental breakdown of plastic pollution, from daily human activity such as tire wear, and fibers shed from synthetic clothing and textiles (Thompson et al., 2004). The global burden of plastic waste in the environment is projected to more than double in the next 30 years to 12 billion tons by 2050 (Geyer et al., 2017). MPs have been found on the highest peaks of Mount Everest (Napper et al., 2020), the deepest trenches of the oceans (Peng et al., 2018), and the most remote locations of the western United States (Brahney et al., 2020), and arctic (Ross et al., 2021).

Humans are exposed to MPs in both bottled and tap water (Kosuth et al., 2018; Madejski et al., 2020), contaminated food sources including vegetables (Oliveri Conti et al., 2020), honey (Diaz-Basantes et al., 2020), beer (Diaz-Basantes et al., 2020; Kosuth et al., 2018), tea (Hernandez et al., 2019), milk (Diaz-Basantes et al., 2020), salt (Kosuth et al., 2018), sugar (Afrin et al., 2022), seafood (Rochman et al., 2015; Van Cauwenberghe and Janssen, 2014), and meat (Kedzierski et al., 2020), as well as outdoor and indoor air (Akhbarizadeh and Dobaradaran, 2021; Bank and Hansson, 2019; Ageel et al., 2022; Brahney et al., (2020), 2021; Dris et al., 2017. Given that humans spend 70–90 % of their time indoors (Klepeis et al., 2001; Soltani et al., 2021, 2022), understanding the microplastic pollution from the indoor environment is particularly important. One convenient measure of this is the analysis of the MP content of household settled dust (Dris et al., 2017; Soltani et al., 2021, 2022), which predominantly comprises cellulosic and polymeric fibers including MPs shed from textiles such as carpet and clothing (Soltani et al., 2021, 2022).

The progress in understanding MP pollution and its consequences for health is severely limited by the difficulty of isolating and identifying MPs from environmental samples. Working to address these challenges by a variety of methods, the field has yet to establish standardized materials and protocols with demonstrated reproducibility. The capture of microparticles and subsequent analyses for the detection of MPs involve passing samples through sieves or other filters to remove macroscopic debris (Kosuth et al., 2018; Maes et al., 2017), followed by digestion of biologicals and organics, typically through oxidation (Green et al., 2018) and/or alkaline buffers (Maes et al., 2017). Finally, MPs are captured on membrane filters. Because the filters used are often incompatible substrates for multiple analyses (i.e., optical microscopy followed by spectroscopy) (Kosuth et al., 2018; Maes et al., 2017), manual transfer to an inorganic substrate is necessary to identify MP polymer type via Raman or Fourier-Transform Infrared (FTIR) spectroscopies. We have observed manual transfers to increase the likelihood of bias, contamination, and loss, and further limits analyses to MPs that are large enough to manipulate by hand. Furthermore, Nile Red is often utilized to stain for plastics (Erni-Cassola et al., 2017; Shim et al., 2016); however, as a lipophilic and hydrophobic stain, biological and organic molecules can also be stained (Greenspan et al., 1985). Thus, additional particulates may be introduced as false positives in the context of MPs and skew MPs quantification results. These non-ideal workflows create multiple bottlenecks and reliability issues and help explain the vast disparities in MP quantification seen in the literature (Koelmans et al., 2019).

In this study, we present a novel timesaving and streamlined analytical workflow we call SNAP (silicon nanomembrane analysis pipeline) for the detection and analysis of environmental MPs and demonstrate its application for the analysis of MPs in household settled dust. SNAP enables three sequential analytical steps following particle capture onto size selective silicon nanomembranes: 1) sample staining followed by optical microscopy and image analysis, 2) identification via micro-Raman spectroscopy, and 3) an end-point analytical method, one which may destroy the sample particles, such as SEM. We show that when ordered in this way, it is possible to conduct a multimodal analysis of individual MPs captured on the membrane without the need for particle transfer. We further introduce the use of Trypan Blue as a counterstain for Nile Red to colorimetrically identify cellulosic fibers. We demonstrate SNAP by sampling and analyzing household settled dust for the presence of MPs.

## 2. Materials and methods

### 2.1. Production of reference microplastic particles

Cryomilled plastic particles were generously gifted to us by Dr. Kim Rogers at the EPA. Briefly, virgin plastic polymer pellets were purchased in the Polymer Kit 1.0 from Hawai’i Pacific University (HPU) Center for Marine Debris Research and contain the most common manufactured polymer types (Geyer et al., 2017). In this study we selected six common polymer types to use as reference plastic materials: low density polyethylene (LDPE), high density polyethylene (HDPE), polystyrene (PS), polypropylene (PP), polyethylene terephthalate (PET), and polyamide (PA). Polymer pellets were used as feed stocks for milling using a Retsch ball cryomill in 5 grind cycles, each at 25 hz for 2 minutes/cycle with a 30 second intermittent cooling cycle at 5 hz. The resulting milled powder was dispersed in water with (0.5 % v/v) surfactant (FL-70; Fisher Scientific, SF105–1) in a glass vial and bath sonicated for one hour prior to vacuum filtration using a 1 μm PTFE filter. Retentate mass was measured, and particles were stored in solution with ultra-pure water and 0.5 % v/v FL-70 to a concentration of 50 g/L. To minimize contamination, all powders were handled in a NanoHood and only metal utensils were used and triple-rinsed with ethanol between uses.

Environmentally representative, or weathered, plastic particles were also produced. Weathered plastics were collected from a marine environment in the Pacific Ocean by Dr. Jennifer Lynch with National Institute of Standards and Technology (NIST)/HPU and were used as feedstocks for milling as described above. Weathered plastics were classified as either mildly or severely weathered as previously described (Brignac et al., 2019). Plastic particle descriptions and concentrations are summarized in Table 1.

### 2.2. Production of reference microfibers

The plastic fibers PET, PP, and PA were purchased from Goodfellow (ES305710, PP305747, and AM325705 respectively). Acrylic and rayon fibers were generously gifted to this research project by Minifibers, Inc., and cotton, linen, and wool/silk blended fibers were purchased through Paradise Fibers (ECT201–04, PFFL-T151–04 and B120, respectively). Using a common method first introduced by Cole et al. (Cole, 2016) utilizing a cryomicrotome the fibers were prepared to a 3:1 (*l:w*) aspect ratio to maintain consistency and determine fiber lengths within the instruments size limitation of all fibers. Fiber characteristics are summarized in Table 2. Briefly, fibers were wrapped around a spool, Epredia Neg-50 freezing solution (VWR, 84000–156) was applied and allowed to freeze at −80°C (~ 10 minutes). Frozen fiber sections were sliced using a Leica CM1860 UV Cryomicrotome at determined lengths based on aspect ratios (limited to 100 μm), thawed at 70 °C, washed with ultrapure water to dissolve glycol, and ultracentrifuged (Beckman Coulter Optima TLX) at 50,000 rpm for 5 minutes at 4 °C (to prevent heating of centrifuge and thus the polymers in the sample) to pellet the fibers and remove the glycol. The pellet was washed three times with ultrapure water and the plastic fibers were resuspended in ultrapure water with 0.01 % v/v Tween 20 (Cole, 2016) (Promega, H5152) and stored in glass vials.

### 2.3. Silicon nanomembrane analysis pipeline (SNAP)

#### 2.3.1. Particle capture and optical microscopy: nile red and trypan blue staining

**Fig. 1.**
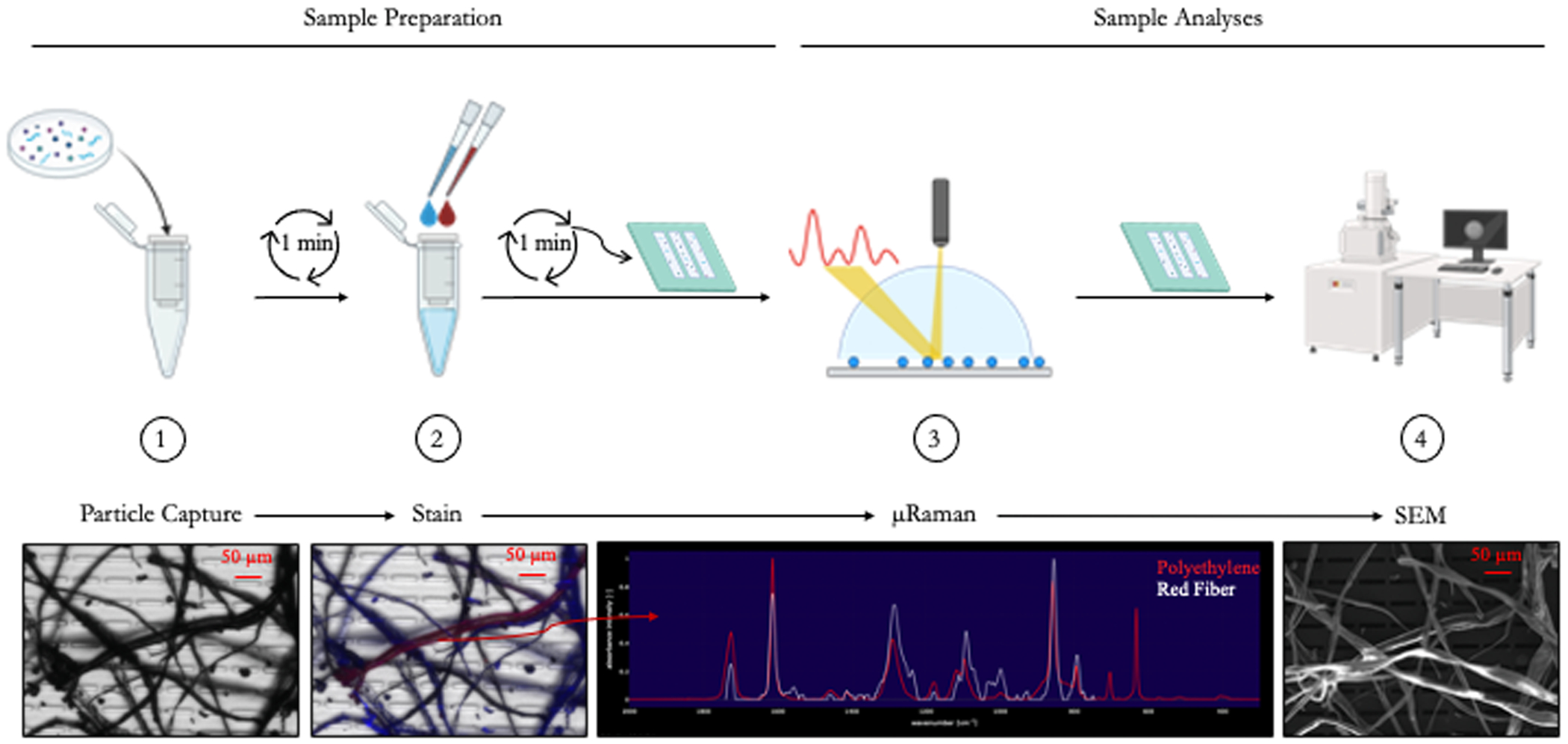
Schematic of Silicon Nanomembrane Analysis Pipeline (SNAP). 1) Particle capture onto silicon nanomembrane. 2) Counterstaining with Nile Red and Trypan Blue for material assessment of particles. 3) Particle identification via Raman spectroscopy. 4) End-point technique, in this example, SEM was performed to characterize particle morphology.

Reference cryomilled particles and reference microfibers were captured for analysis via filtration through a SepCon Vial (SiMPore, Inc., West Henrietta, NY; SC400524) containing a size selective silicon nanomembrane, dependent on size of particles captured (2 μm – 20 μm x 50 μm slit pores, SiMPore, Inc., MSSN400–3L-2.0). SepCon Vials were assembled as described (https://nanomembranes.org/sepcon-assembly-written-description/). An aliquot of 20 – 50 μL (using 10 μL increments) of the prepared reference cryomilled particles (50 g/L) was filtered as described to obtain an even particle dispersion on the surface of the membrane. Fiber concentration was determined using a hemacytometer and 1000 – 5000 fibers were captured onto nanomembranes, obtaining an even particle dispersion. Particle dispersion was observed on the membrane inside of the SepCon device using a dissecting microscope. Captured particles were stained *in situ* (*i.e., immediately following filtration with the same membrane and in the same vial*) with 20 μL of 1 μg/mL Nile Red stain (Abcam ab228553) for 5 minutes followed by three washes with ultrapure water. 500 μL of ultrapure water was added to the SepCon, spun on a tabletop centrifuge at 500 rpm for one minute, and flowthrough was discarded, followed by staining with 20 μL of 0.4 % Trypan Blue (Millipore Sigma) stain for 5 minutes followed by three washes with 500 μL of ultrapure water (Fig. 1). Nanomembranes were carefully removed from the SepCon Vial and allowed to dry in a 70 °C oven for 2–5 minutes. All samples were processed inside a laminar flow fume hood and all reagents were filtered through a 1 μm slit pore nanomembrane to prevent contamination from the workbench and counterstaining protocol, respectively. Nanomembranes with captured particles were housed and transported enclosed in gel boxes provided by the manufacturer SiMPore Inc. to prevent contamination. Stained particles were imaged under a Nikon epifluorescent microscope (Nikon Ti2E, Nikon Corporation, Tokyo, Japan) equipped with a Zyla sCMOS camera (Andor Technology, Belfast, UK) and CoolLED PE-4000 light source. Nile Red fluorescence was detected using the TRITC channel at excitation wavelength of 525 nm and emission wavelength of 595 nm and Trypan Blue fluorescence was detected using the Cy5 channel at excitation wavelength of 635 nm and emission wavelength of 697 nm. Microscopic images were obtained at 4x, 20x, or 40x magnification and saved as separate TIF images. Overlayed images were prepared in imageJ (Fig. 4).

#### 2.3.2. Polymer identification: raman spectroscopy

Raman spectroscopy was performed on captured particles using ~50 mW of near-infrared cw laser (Innovative Photonic Solutions) excitation at 830 nm to reduce the influence of fluorescence noise. A 50X objective in an upright microscope (Nikon Eclipse E400) focused the laser light downwards to a spot diameter of approximately 5 μm. Captured particles on silicon nanomembranes were translated in three dimensions to the laser focus via visible light transillumination guidance. Epi-detected light from the sample plane was long-pass filtered to reject the excitation wavelength and confocally imaged onto a circular bundle of forty multimode optical fibers with core diameters of 100 microns. At the bundle’s other end, the fibers were rearranged into a vertical column in the slit plane of an imaging spectrograph (Kaiser HoloSpec). For each sample, 5 image frames of 10 seconds were acquired via a CCD array thermoelectrically cooled to −50°C (Andor Technology, Belfast UK). After digital processing to remove cosmic ray artifacts and spatial distortion, spectra from all fibers were summed to produce a single vector of spectral intensities. Relative wavenumbers for all pixels were calibrated using neon lamp emission peaks for absolute wavelength followed by acetaminophen Raman peaks for relative wavenumber. A seventh-order polynomial was digitally subtracted from the raw data to suppress broad features from fluorescence and emphasize the Raman peaks. Calibrated spectra were identified with OpenSpecy, an open source Raman spectroscopy database that includes 636 spectra from three different libraries of 276 polymers and materials applicable to identifying environmental microplastics and fibers (www.openanalysis.org/openspecy) (Cowger et al., 2021).

Raman spectroscopy was performed on an n = 3 of each NR, TB, and unstained particles and fibers per household dust sample (where applicable, some samples contained less than 3 of each of these particles and fibers as with household dust sample 2 and 3, shown in Fig. 4). Representative Raman spectra results of one plastic polymeric particle and fiber, one cellulosic polymeric particle and fiber, and one proteinaceous/inorganic particle and fiber are included in Supplemental Table 1.

#### 2.3.3. End-point method: SEM

The same captured particles were then prepared for Scanning Electron Microscopy (SEM) following Raman spectroscopy by coating a thin layer of Au (6 nm thick, 100–150 mTorr, 15 mA, 60 s, Denton Prep Sputtering System). SEM images of particles were obtained on a Zeiss Auriga SEM.

### 2.4. Household settled dust sample collection

Household settled dust was collected from three different households in Rochester, NY in March 2022. Glass petri dishes (90 mm) with glass lids were placed at a height of 3 – 4 feet in a central location in the household, avoiding areas with food cooking, candles, or near open windows. The bottom glass petri dish (dust collection dish) was placed open faced to allow collection of settled dust for 7 consecutive days. The glass lid was placed face down to avoid dust collection. After the stated collection period, the dishes were wrapped in aluminum foil for transport to the laboratory. Petri dishes were rinsed three times with 1mL ultrapure water and filtered through a SepCon Vial and collected debris was processed as described via SNAP. A dust negative control was obtained by placing a glass petri dish in a clean room (HEPA filtered, rated at 1000 particles per cubic foot) at the University of Rochester Department of Biomedical Engineering and analyzed as described. A methods control was obtained by running a blank silicon nanomembrane through all methods simultaneously during sample processing to assess contamination produced from the protocol.

## 3. Results and discussion

### 3.1. Discussion of methods used in SNAP

Silicon nitride nanomembranes were used in this study for their demonstrated utility for the capture and characterization of MPs. We previously demonstrated the use of silicon nanomembranes for the rapid isolation and concentration of MPs from municipal drinking water followed by *in situ* numeration using optical microscopy (Madejski et al., 2020). The lack of particle transfer steps minimizes bias, loss, and contamination arising from repeated handling, and the relatively small active membrane area of the highly permeable nanomembranes localizes the materials of interest to a small field of view and speeds up analysis. We have further showed that silicon nanomembranes outperform commercially available filters commonly used for MPs analysis, such as polycarbonate and polytetrafluoroethylene, in several aspects including porosity and background reduction in optical microscopy (Carter et al., 2023). Silicon nanomembranes were originally developed at the University of Rochester and are extremely thin (15nm) and highly permeable (Striemer et al., 2007). The advantages of using these silicon nanomembranes include: 1) are orders-of-magnitude more permeable than conventional membranes; 2) have uniform, precisely patterned pores enabling precise separations; 3) provide outstanding transparency for optical imaging, 4) provide negligible or low-interference background signals in optical and electron microscopy and spectroscopic analysis; and 5) are inert to chemicals and temperatures that can easily damage polymeric membranes. These attributes make silicon nanomembranes outstanding substrates for the capture and subsequent *in situ* analyses of MPs.

Cryomilling plastics has become a popular method to produce MP fragments, morphologies that closely resemble those found in the environment, and in the size range from nano- to micrometer sized particles. Due to instrumental limitations, the accurate polymer identification of MPs via Raman spectroscopy is limited to particles greater than one micron (Tewari et al., 2022). The cryomill is an ideal instrument to produce secondary MPs (plastics formed by the breakdown of larger plastics or primary plastics) as it grinds down primary plastics in the presence of liquid nitrogen, thereby, preventing the polymer from heating up or melting during the process, which would ultimately change the particle morphology. Many publications have cited the use of this method to create MP reference materials (Jungnickel et al., 2016; Scircle et al., 2020; Tewari et al., 2022). Thus, we relied on this technique to produce reference MPs to use as control particles in our methods development.

Nile Red (9-diethylamino-5H-benzo[α]phenoxazine-5-one) is a hydrophobic and lipophilic dye that is a highly utilized stain in the MPs field but has historically been used to stain lipids, and proteins in the beta-sheet conformation, in biological samples. This creates a challenge when trying to identify MPs in various environmental samples, especially biological samples, as there may be other hydrophobic and lipophilic environmental substances present in the sample. Studies have found that fibers account for more than 90% of household settled dust (Dris et al., 2017; Sharifi Soltani et al., 2022; Soltani et al., 2021) and natural cotton and synthetic polyester fibers make up the two most common fiber types used in textiles and clothing. To distinguish plastics from other biological materials, including cellulose, we utilized a counterstain using Trypan Blue. Trypan Blue is known for its application in cell viability assays as it stains dead cells; however, the blue dye also has a strong binding affinity for cellulose. In this study, we tested the counterstain of Nile Red with Trypan Blue to distinguish plastics and cellulosic particles from other materials found in household settled dust. Cole first described a method to produce reference microfibers and this protocol has been highly utilized in the MPs field (Cole, 2016; Sait et al., 2021; Song et al., 2022). Due to the fibrous nature of household settled dust, we produced reference microfibers of various fiber types including plastic polymers (PP, PET, Nylon, and Acrylic), cellulosic polymers such as cotton and linen, and proteinaceous fibers including wool and silk. Rayon is a reconstituted cellulosic fiber, which is a synthetic polymeric cellulose.

Household settled dust analyzed in this study primarily consisted of natural and petrochemical-based synthetic fibers, consistent with previous findings (Sharifi Soltani et al., 2022; Soltani et al., 2021). While organic matter digestion using oxidizing agents can help eliminate potential false positives, such as lipid interference with Nile Red staining, this step was not included in our procedure. Instead, SNAP incorporates Raman spectroscopy to confirm and rule out false positives. Organic digestion is more critical for samples with substantial organic content, such as biological tissues or environmental water, where organic matter could interfere with microplastic identification. Given the composition of household dust, digestion was deemed unnecessary for accurate analysis in this context.

Raman spectroscopy is more versatile than infrared (IR) spectroscopy, especially with respect to material identification in biological samples. Raman spectroscopy is a scattering light technique, making it able to analyze smaller samples more easily than IR, an absorptive technique that necessitates larger specimens and more constraints on sample placement (Kerr et al., 2015). Furthermore, the substrates used for infrared spectroscopy usually require either silver or gold-coating to enhance reflectance. It has been previously suggested that substrates for Raman spectroscopy containing silicon or silicon oxide can yield reliable results with minimal background noise (Mikoliunaite et al., 2015). Nile Red fluorescence of particles is a concern with Raman spectroscopy analysis as it may add significantly to background noise. In this study, however, we counterstained polymers with Nile Red and Trypan Blue prior to Raman spectroscopy with an 830nm laser and little noise was observed (Supplemental Table 1). In the SNAP procedure, staining and optical microscopy can be performed either prior to or after Raman spectroscopy, depending on the Raman system of use. However, the objective of counterstaining is to save time by only analyzing Nile Red stained particles for MPs analyses.

### 3.2. Development of SNAP using reference materials

The SNAP workflow for reference materials is graphically outlined in Fig. 1. Cryomilled virgin particles were captured onto 20 μm pore membranes, weathered cryomilled particles were captured onto 8 μm x 50 μm slit pore membranes, and either 2 or 8 μm x 50 μm slit pore membranes were used to capture microfibers. The nanomembranes used in this study demonstrate the versatility of silicon nanomembranes with different pore sizes and shapes for isolating particles. Depending on the size of the particles of interest, different membranes can be selected to match instrument limitations, such as Raman spectroscopy (10 μm) and FTIR (20 μm). SiMPore, Inc., produces microporous membranes with circular pores (0.5 μm and 20 μm) and microslit membranes with elongated pores (0.5, 1, 2, 4, and 8 μm). The pore shape and porosity influence sample filtration efficiency, enabling tailored applications, such as isolating particles from large volumes of water or achieving even particle dispersion.

**Fig. 2.**
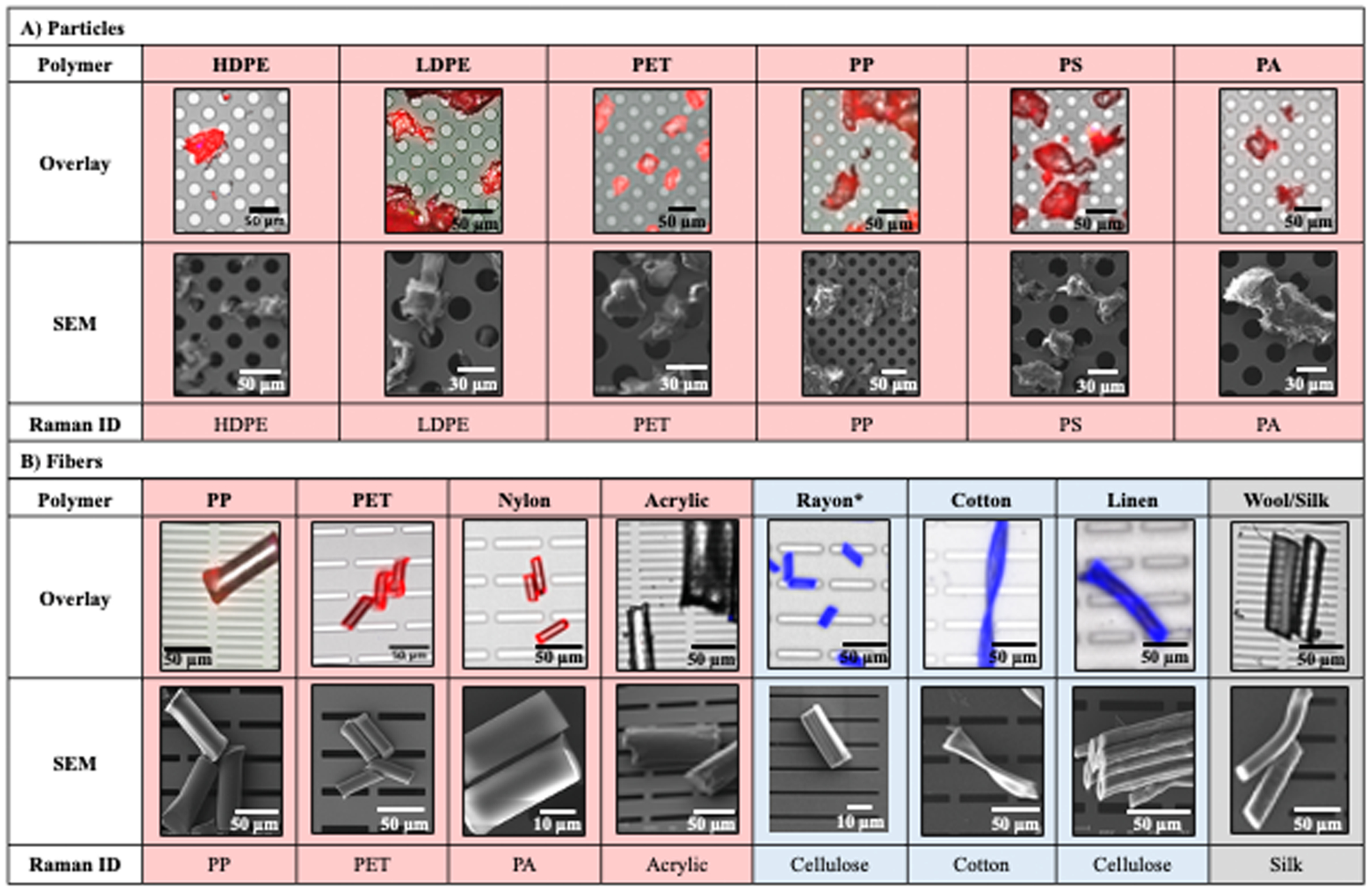
Characterized reference MPs. A) Cryomilled reference MPs (20 – 100 μm) captured on silicon nanomembranes with 20 μm circular pores. B) Reference microplastic fibers (fiber diameters 8–42 μm) captured on silicon nanomembranes with 8 × 50 μm slit pores (PP, Acrylic, and Wool/Silk membranes include 2 ×50 μm slit pores). Overlay: overlaid fluorescent images of Nile Red and Trypan Blue stained particles. SEM: SEM images of stained particles. Raman ID: Raman spectroscopy polymer identification. Materials characterized as plastic polymer (false colored in red), cellulosic (false colored in blue), and proteinaceous/inorganic (unstained, greyscale). *Reconstituted cellulose.

In Fig. 2, a 20 μm microporous nanomembrane was used to capture cryomilled particles with a size range of 20–100 μm, ensuring even dispersion. For the analysis of rayon fibers (8 μm in diameter), an 8 μm microslit nanomembrane was used, successfully capturing fibers as shown in the figure. Importantly, the differences in nanomembrane pore size or shape did not impact the particle characterization or identification results.

After particle capture, *in situ* analyses and optical microscopy was performed, including staining. All cryomilled particles and fibers were counterstained with Nile Red and Trypan Blue. We show that the six most common manufactured virgin plastics (20 – 100 μm HDPE, LDPE, PET, PP, PS, and PA) take up the Nile Red stain and not the Trypan Blue stain after cryomilling (Fig. 2A). Additionally, all plastic polymer fibers took up the Nile Red stain and not the Trypan Blue stain, with the exception of Acrylic, which remained unstained (Fig. 2B, red), as has been previously shown (Stanton et al., 2019). All cellulosic fiber types took up the Trypan Blue stain, but not the Nile Red stain, as expected, including rayon (Fig. 2B, blue). The proteinaceous fibers, wool and silk, remained unstained, as expected (Fig. 2B, grey). These results suggest counterstaining Nile Red with Trypan Blue is a reliable colorimetric method for distinguishing plastic fibers from other natural and proteinaceous materials found in environmental samples. However, Stanton et al. (2019) reported Nile Red staining of dyed fibers such as rayon, silk, wool, and polyamide, which they attributed to the dyes used in the coloring process (Stanton et al., 2019). Here, we used minimally processed, undyed fibers to avoid interference from additives or dyes. While this approach is not representative of the dyed fibers commonly found in environmental samples, it allowed us to evaluate the staining properties of pristine polymeric fibers. Our findings show that pristine plastic fibers (except acrylic, consistent with Stanton et al.) take up NR stain, cellulosic fibers take up TB stain but not NR, and proteinaceous fibers take up neither stain. Furthermore, we demonstrate that neither Nile Red or Trypan Blue staining of particles contribute to the measured size of the particles (Supplemental Figure 4).

Once plastics are identified with Nile Red staining and fluorescent imaging, the next steps of SNAP can be performed. First is particle identification via non-destructive Raman spectroscopy. We demonstrate the successful Raman identification of various polymers collected onto silicon nitride nanomembranes. Despite Nile Red staining earlier in the SNAP workflow, Raman spectroscopy accurately identified each polymer type of the cryomilled particles and microfibers (Fig. 2; Supplemental Table 1).

The final step of SNAP terminates the sequence of analytical methods with scanning electron microscopy (SEM). SEM is considered an end-point technique because it requires sample preparation in the form of a thin metal coating to prevent charging in the electron beam. SEM reveals particle morphology and can be performed in combination with energy-dispersive X-ray spectroscopy (EDAX) for concurrent elemental analysis on some electron microscopes (Madejski et al., 2020). SEM images of the plastic fibers demonstrated a smooth surface morphology, likely due to the process of extrusion during fiber production (Fig. 2B, red). Cellulosic fibers demonstrated either a spiral (cotton) or tubular shape (Fig. 2B, blue), while proteinaceous fibers displayed more of a string-like or scaly surface (Kozłowski and Mackiewicz-Talarczyk, 2020; Puchowicz and Cieslak, 2022) (Fig. 2B, grey). To address the complexity of environmental samples, we emphasize the importance of using multiple analytical methods. We recommend the SNAP approach, which combines staining, spectroscopic analysis, and SEM characterization, to ensure accurate microplastic detection in environmental samples.

To further explore the analytical methods of SNAP, we also evaluated weathered MPs. Plastic pollution in the environment is exposed to ultraviolet (UV) radiation, which causes embrittlement, forming MPs. During the weathering process, UV radiation breaks chemical bonds in the polymers resulting in oxidation of the material, a process known as photodegradation (Romera-castillo et al., 2023). Mildly or severely weathered plastics were obtained from the Pacific Ocean (Brignac et al., 2019) and cryomilled to produce weathered MPs to be analyzed via SNAP. Images of these cryomilled microplastics are shown in Supplemental Figure 1B.

**Fig. 3.**
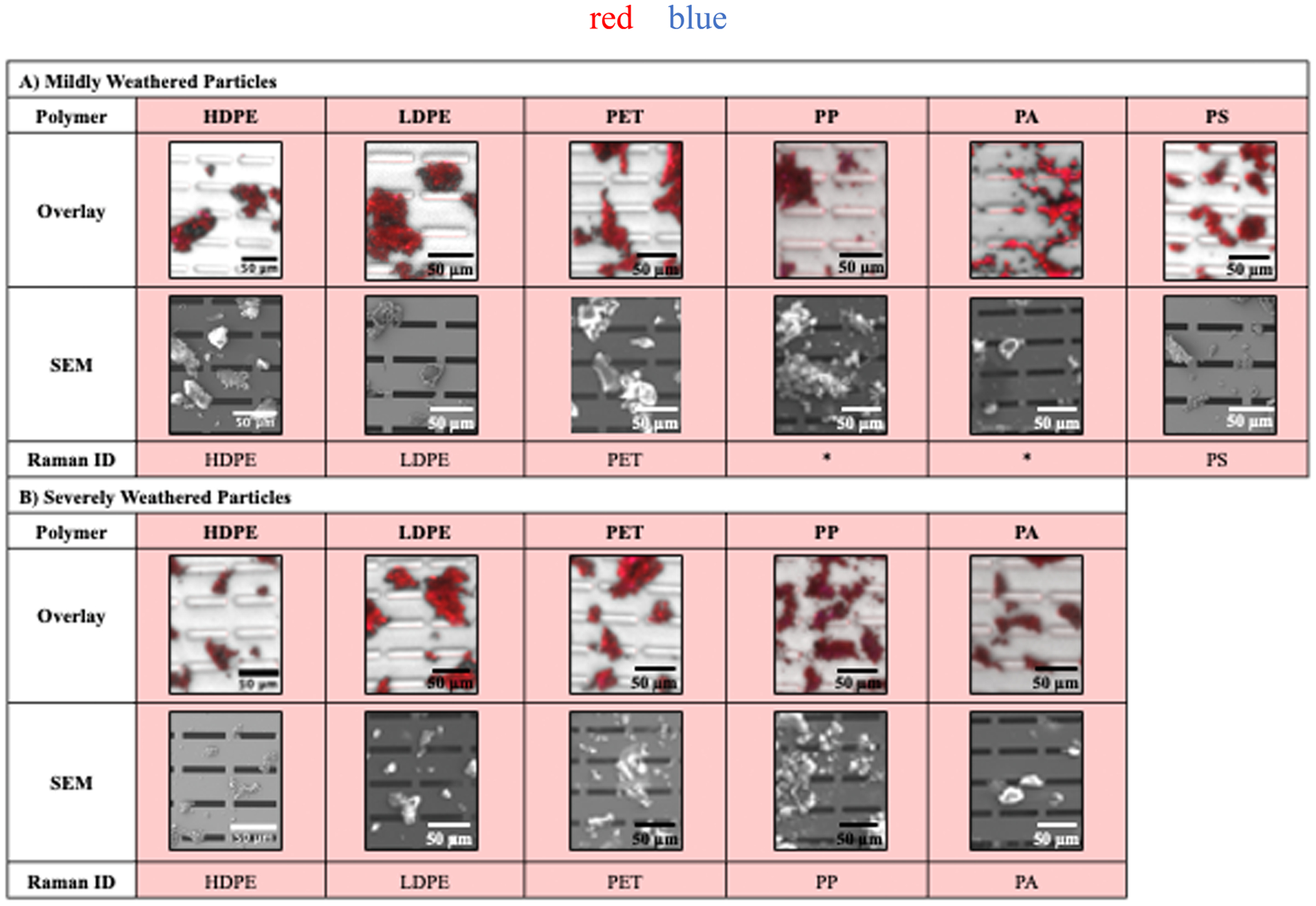
Characterized weathered MPs. Cryomilled weathered MPs (8 – 100 μm), classified as A) Mildly weathered or B) Severely weathered, captured onto silicon nanomembranes with 8 × 50 μm slit pores. Overlay: overlaid fluorescent images of Nile Red and Trypan Blue stained particles. SEM: SEM images of stained particles. Raman ID: Raman spectroscopy polymer identification. Materials characterized as plastic polymer (false colored in red), cellulosic (false colored in blue), and proteinaceous/inorganic (unstained, greyscale). *Raman spectroscopic identification not obtained, see Supplemental Table 1 for details.

We demonstrated that oxidized plastics (see below) still stain with Nile Red stain, but not Trypan Blue (Fig. 3). The colorimetric assessment of all reference particles are shown in Supplemental Figure 3. The cryomilled weathered plastics seem to have smaller fragments present in the sample, likely caused by the oxidation state of the plastics, as compared to the pure polymer pellets, despite using the sample cryomilling protocol. Images of the tested weathered plastic products obtained from the Pacific Ocean prior to cryomilling are shown in Supplemental Figure 2.

Photodegradation of plastics in the environment results in severe weathering including fissure and pore formation on the oxidized surface. We examined this behavior using microplastics from the Pacific Ocean’s Great Pacific Garbage Patch, home to trillions of pieces of oceanic microplastics (Moore et al., 2001). These microplastics were collected by Algalita Marine Research & Education and obtained from their Debris Science Investigation Kit. A blue plastic fragment (2 mm x 3 mm x 2 mm), identified as PE via Raman Spectroscopy, was selected for surface morphology characterization. The fragment was cut in half using a clean razor blade to expose the non-oxidized interior surface of the fragment. SEM imaging analysis revealed pore and fissure formation on the oxidized surface of the blue fragment (Supplemental Figure 5A), which is absent on the interior surface of the fragment (Supplemental Figure 5B). Thus, to produce environmentally relevant microplastics, weathering of the reference materials should be performed after cryomilling. This can be accomplished by performing artificial UV oxidation of the cryomilled MPs.

### 3.3. SNAP application to household dust

**Fig. 4.**
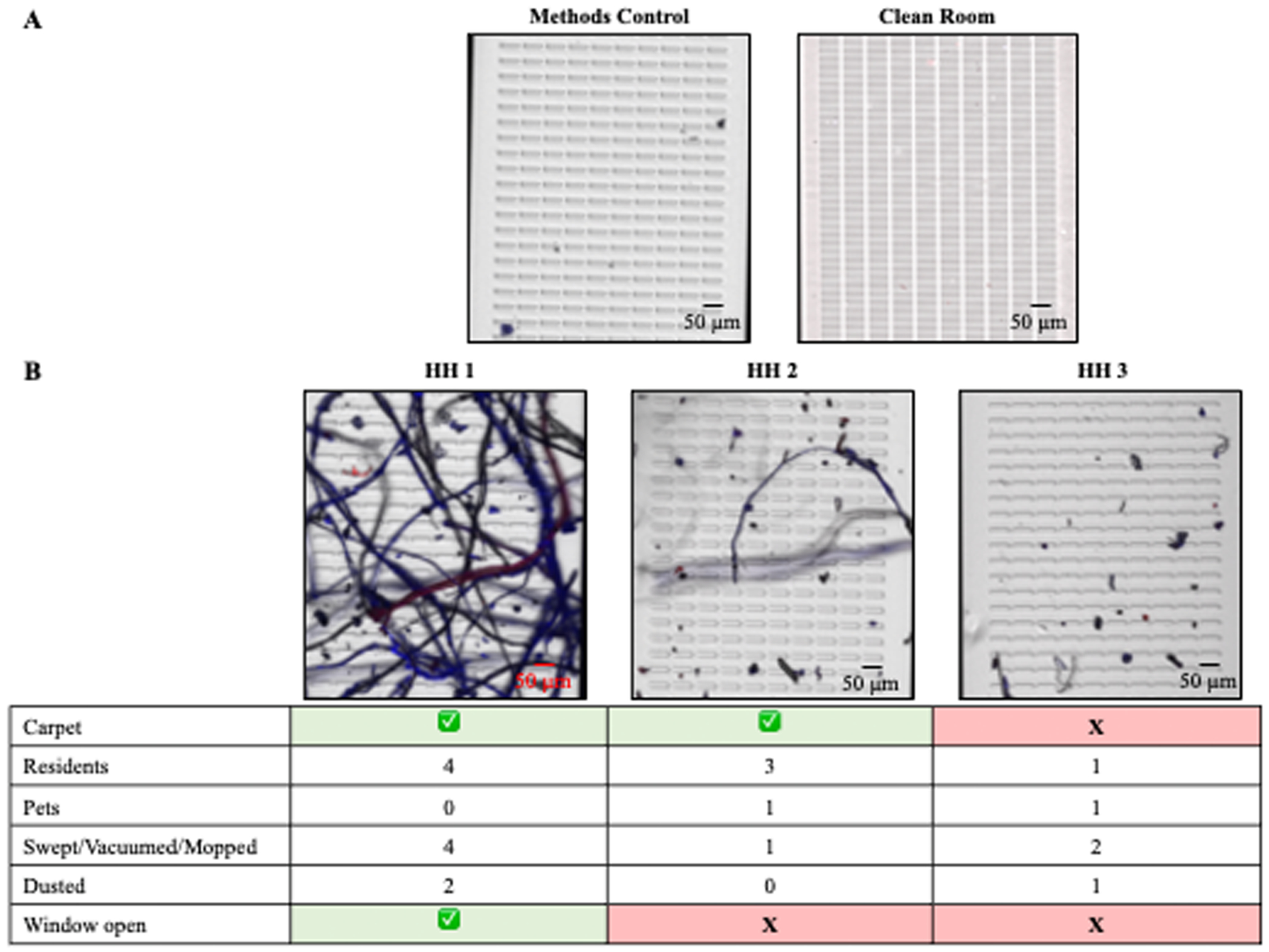
Material assessment of household settled dust. A) Fluorescent overlaid images of counterstained methods and clean room controls and household (HH) settled dust particles collected from three different households captured onto silicon nanomembranes. B) Summarized household questionnaire data.

We tested the applicability of SNAP for identifying particles found in indoor household settled dust samples in households in the Rochester, NY area. A questionnaire was completed by each participating household to provide insight about possible sources of the settled dust (Supplemental Materials, Appendix A). Fig. 4 summarizes the analysis of the collected settled dust from three households. A clean room (HEPA filtration, operating at 100–200 particles/ft^3^, rated for 1000 particles/ft^3^) control was used to assess contamination during dust collection, and showed little contamination occurring during the dust collection procedure. Smaller particles are visible on the clean room control and some particles did take up the Nile Red or Trypan Blue stain; however, these particles do not lie within our detectable limits for polymer identification (Fig. 4A). Our methods control shows few Trypan Blue stained particles. These particles are likely sediments from the Trypan Blue reagent, which can still occur post filtration; however, there were no Nile Red stained particles present in the methods control (Fig. 4A).

In Household One (HH 1) we noticed a significant number of fibers. This household had four residents and cleaned the most within the week of settled dust collection (Fig. 4B). Previous studies have shown that wet mopping and dusting reduced the number of MPs in household settled dust. Due to the lack of specificity of cleaning style (i.e., wet vs dry) in the questionnaire, we could assume this household dry dusted, which could disturb settled dust. Furthermore, this household was the only household that opened a window during the dust collection period, suggesting that outdoor windblown dust may have contributed to the sample. We believe the cleaning style and opening a window contributes most to the fibrous nature of household one as compared to household two. This household also has carpet in the room of dust collection with three residents and a pet animal (not specified); however, Household Two (HH 2) performed minimal cleaning and did not open any windows during dust collection. Household Three (HH 3) demonstrated the least amount of particles captured with only one resident and one pet animal (unspecified), no carpeting, and did not open any windows (Fig. 4B). Households with carpet in the room of settled dust collection seem to have more fibers present in their settled dust samples, which is consistent with findings in the literature (Dris et al., 2017; Soltani et al., 2021, 2022).

**Fig. 5.**
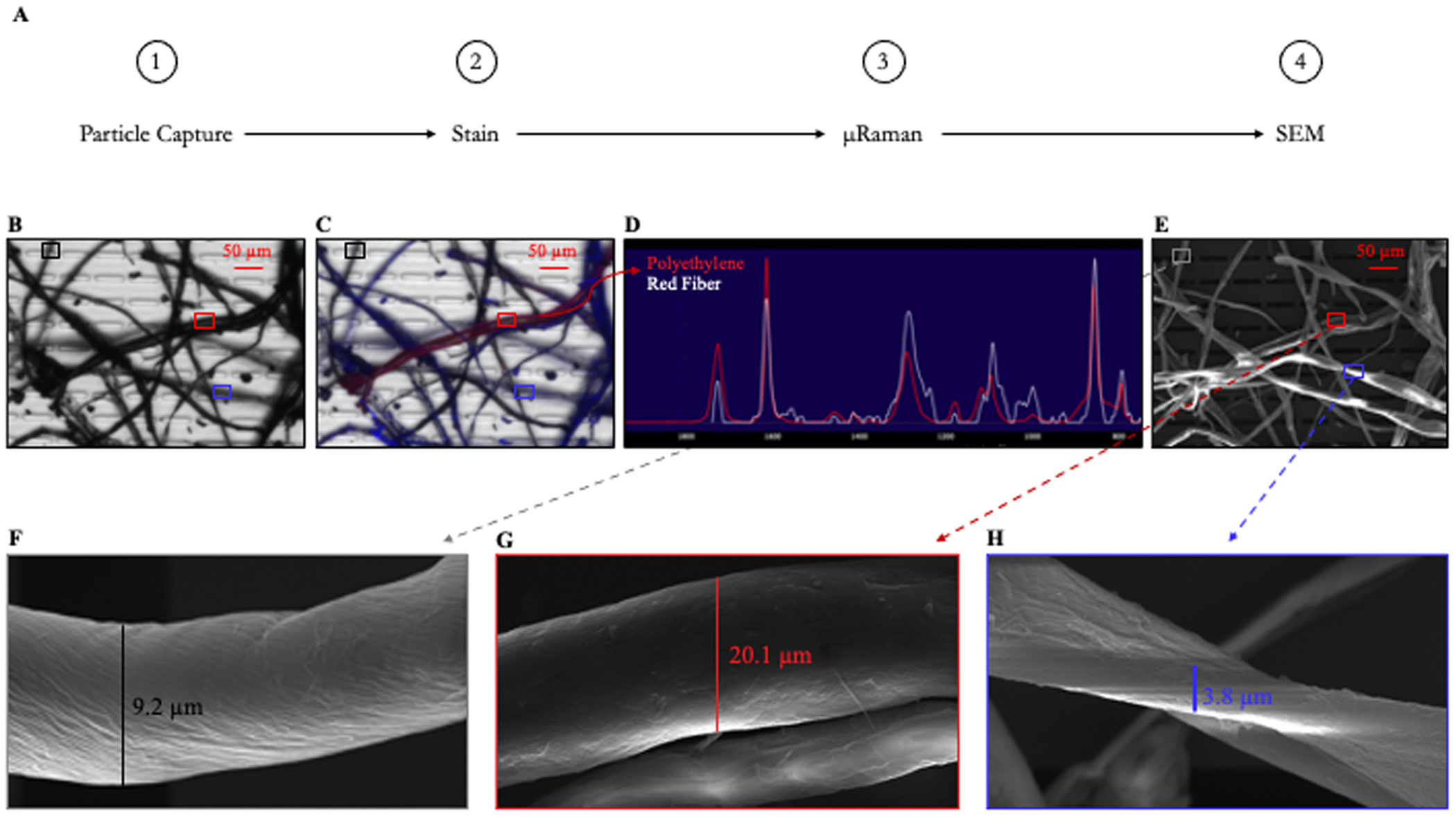
Household Settled Dust Analyzed via SNAP. A) SNAP procedure. B) Greyscale image and C) Overlaid fluorescent image of counterstained household settled dust collected from HH 1. D) Raman spectrum identifying Nile Red stained fiber collected from HH 1 as polyethylene (white spectrum). E) Electron micrograph of household settled dust collected from HH 1. F) Electron micrograph of an unstained fiber collected from HH 1, identified in panels B and C with a black box and in panel E with a grey box. G) Electron micrograph of a Nile Red stained fiber collected from HH 1, identified in panels B, C, and E with a red box. H) Electron micrograph of a Trypan Blue stained fiber collected from HH 1, identified in panels B, C, and E with a blue box.

We further examined the dust collected in HH 1. We observed many Trypan Blue stained fibers and unstained fibers (black), but we only observed one fiber that took up the Nile Red stain (Fig. 5C). Raman spectroscopy identified this fiber as polyethylene (Fig. 5D). Using electron microscopy (Fig. 5E), we examined the structure of these fibers. As seen with our reference materials, the polyethylene fiber has a smooth surface (Fig. 5G). Noticeably absent were the pits and fissures found on the surface of polyethylene sourced from the outdoors (Romera-castillo et al., 2023; Sait et al., 2021), indicating that this fiber was not exposed to weathering conditions.

Many of the Trypan Blue stained fibers have spiral structures, similar to our cotton reference fibers (Fig. 5H). Similarly, the surface morphology of the unstained fibers has a scaly surface (Fig. 5F), which is consistent with our proteinaceous reference fibers. These results suggest that counter staining with Nile Red and Trypan Blue and examining surface morphology via SEM are simple methods that can be used to distinguish plastics from other types of materials found in the indoor environment. We did not quantify total MPs present in household dust samples, as this study was mainly focused on the methods to identify and characterize MPs found in environmental samples while validating the suitability of silicon nanomembranes for MPs research.

## 4. Summary

Here we advance the utility of silicon nanomembranes for the detection and analysis of indoor MPs and other particulates within household settled dust with a novel, timesaving, and multimodal analytical workflow we call SNAP. In SNAP, particles are captured onto silicon nanomembranes and analyzed *in situ* by allowing direct correlation between multiple modalities applied to the same sample. The SNAP workflow eliminates the need for lossy manual transfers and supports the characterization of smaller MPs found in environmental samples. We also introduce the novel use of Trypan Blue as a counterstain with Nile Red stain to more clearly distinguish MPs from other biological and cellulosic materials found in environmental samples. Finally, we demonstrate the use of SNAP as a proof-of-principle application for the analysis of household settled dust.

## Supplementary Material

MMC1

MMC2

MMC3

MMC5

MMC4

MMC6

Supplementary data associated with this article can be found in the online version at doi:10.1016/j.eti.2025.104106.

## Figures and Tables

**Table 1:** Polymer Characteristics. Polymer type,source,storage,degree of weathering,particle processing steps, and concentration of particle solutions.

Polymer	Source	Storage	Degree of Weathering	Processing	Concentration (g/L)
**LDPE** **HDPE** **PS** **PP** **PET** **PA**	Polymer Kit 1.0	Dispersed (0.5 % v/v FL–70)	Virgin Plastics	Cryomilled, sonicated, filtered (≥ 1 μm)	50
**LDPE** **HDPE** **PS** **PP** **PET** **PA**	Marine environment in the Pacific Ocean	Dispersed (0.5 % v/v FL–70)	Mild		50
**LDPE** **HDPE** **PP** **PET** **PA**			Severe		

**Table 2:** Fiber Characteristics. Polymer type, manufacturer, product ordering number, physical diameter, and aspect ratio of microfibers produced.

Fiber	Manufacturer	Product Number	Diameter (μm)	3:1 Aspect Ratio (μm)
**PET**	Goodfellow	ES305710	14	45:14 *
**PP**		PP305747	28	90:28 *
**PA**		AM325705	10	30:10
**Acrylic**	Minifibers, Inc.	N/A	42	100:42 *
**Rayon**			8	25:8 *
**Cotton**	Paradise Fibers	ECT201–04	20	60:20
**Linen**		PFFL-T151–04	Varies	100:x
**Wool/Silk**		B120	18	60:18 *

* Indicates deviation from 3:1 ratio based on cryostat limitations.

## Abbreviations:

HH: Household
LDPE: Low Density Polyethylene
HDPE: High Density Polyethylene
MPs: Microplastics
PA: Polyamide
PET: Polyethylene Terephthalate
PP: Polypropylene
PS: Polystyrene
SEM: Scanning Electron Microscopy
SNAP: Silicon Nanomembrane Analysis Pipeline
