# Supplementary material for: Assessment of household settled dust via silicon nanomembrane analysis pipeline (SNAP)
Source: Environ Technol Innov. Author manuscript; Available in PMC 2025 Jun 26. (PMC12201965; doi:10.1016/j.eti.2025.104106)
Supplement: MMC1 [file NIHMS2077742-supplement-MMC1.docx]

**Supplemental Materials**

**Assessment of Household Settled Dust via Silicon Nanomembrane Analysis Pipeline (SNAP)**

Samantha S. Romanick, Gregory Madejski, Garrett Cashion, Andrew J. Berger, Alison Elder, James McGrath

Number of Pages: 4

Appendices: 1

Number of Figures: 3

| **A**  **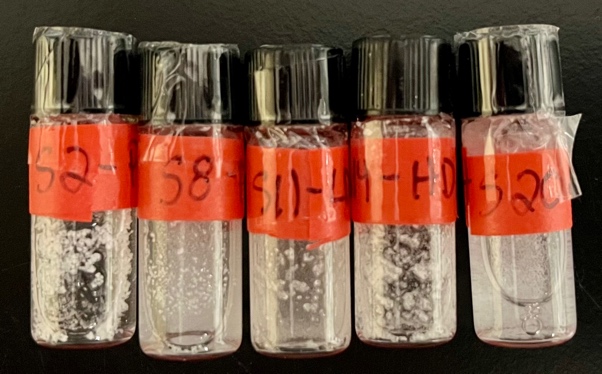** |
| --- |
| **B**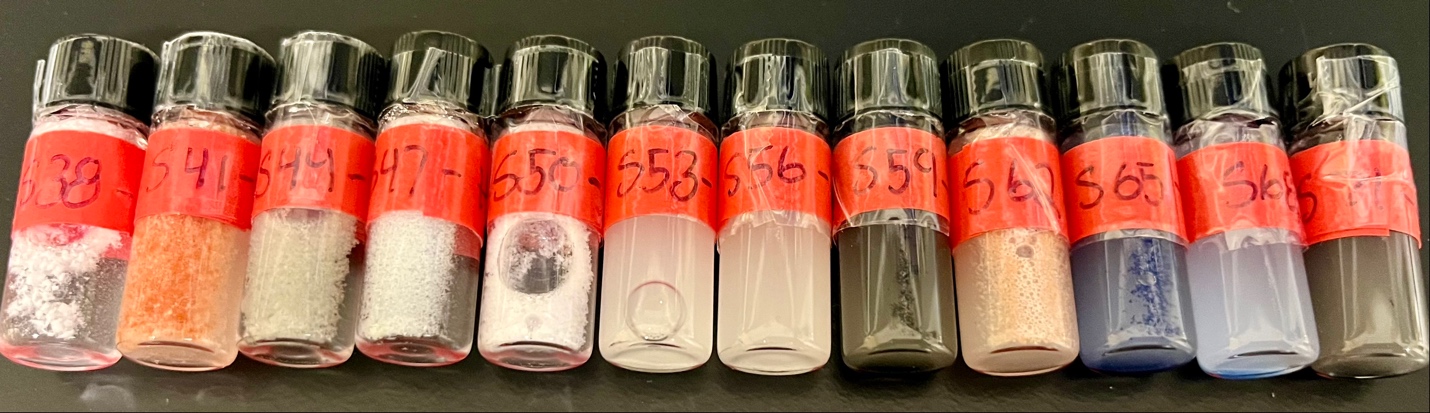 |
| **Supplemental Figure 1.** Photo images of **A**) cryomilled reference microplastic particles (where S2 is PP, S8 is PET, S11 is LDPE, S14 is HDPE, S20 is PA. S5 PS is not shown); and **B**) cryomilled weathered microplastic particles (where S38 is LDPE mild, S41 is LDPE severe, S44 is LDPE fiber mild and not analyzed in this study, S47 is HDPE mild, S50 is HDPE severe, S53 is PET mild, S56 is PET severe, S59 is PP mild, S62 is PP severe, S65 is PA mild, S68 is PA severe, and S71 is PS mild). |

| **Polymer** | **Mild** | **Severe** |
| --- | --- | --- |
| HDPE | 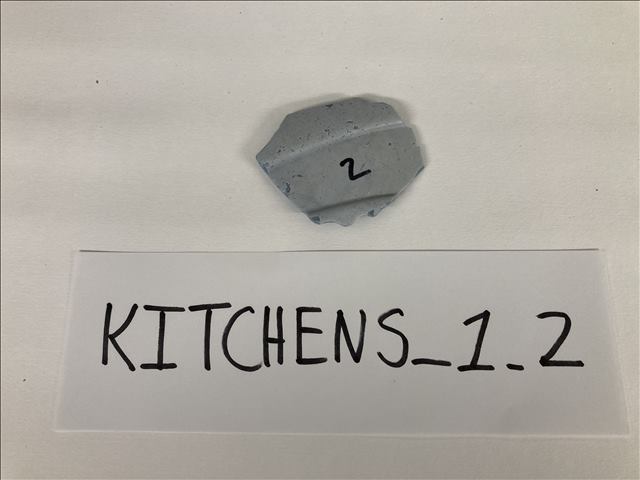 | 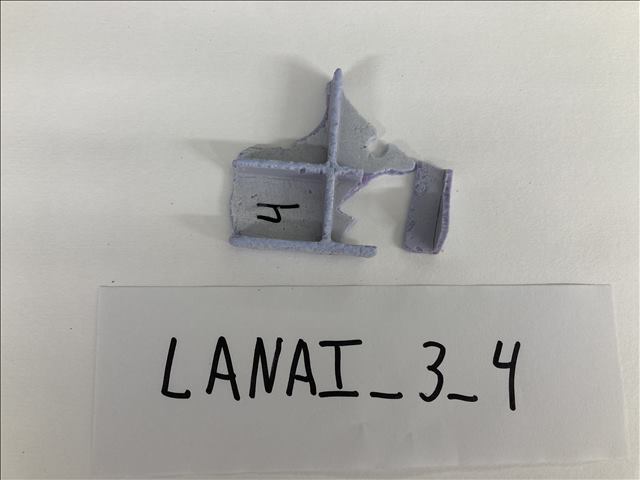 |
| LDPE | 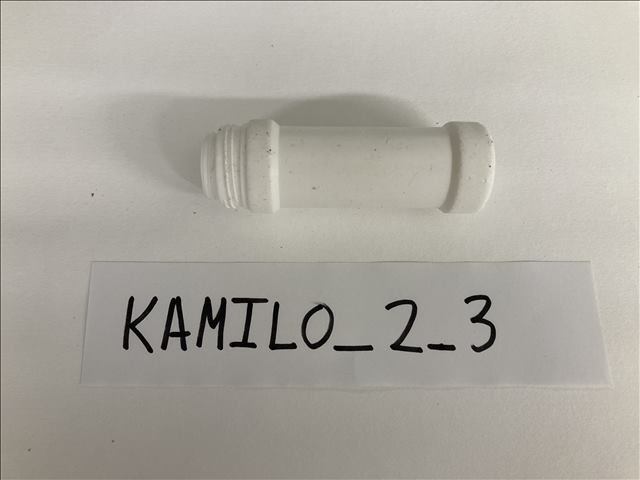 | 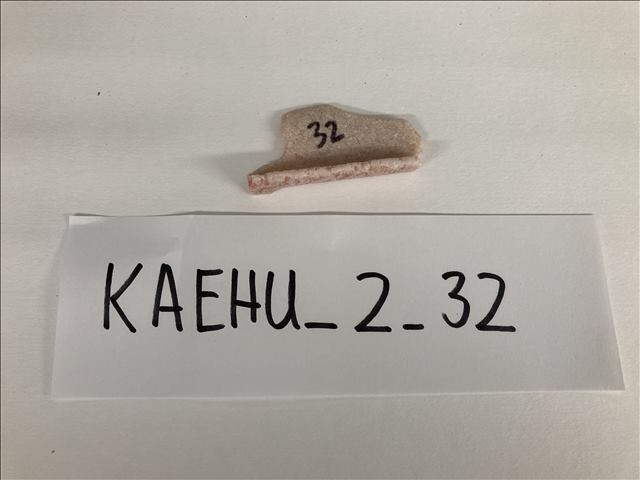 |
| PET | 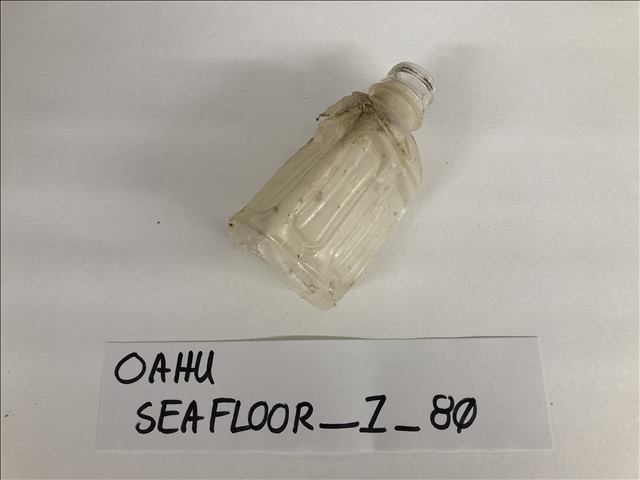 | 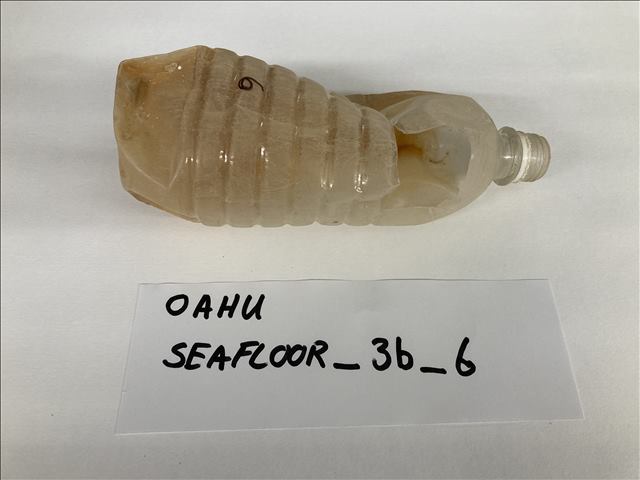 |
| PP | 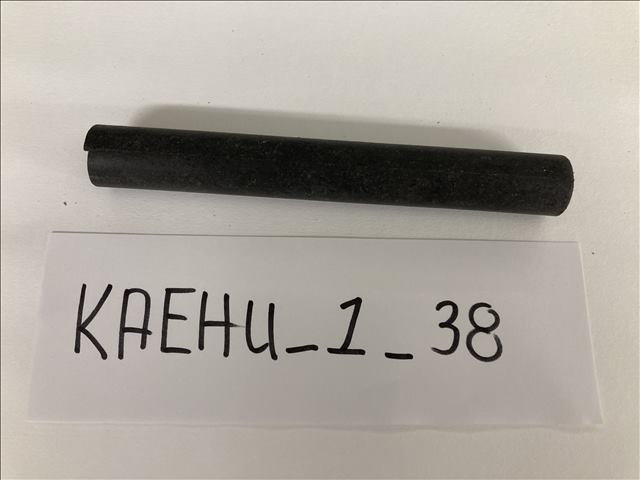 | 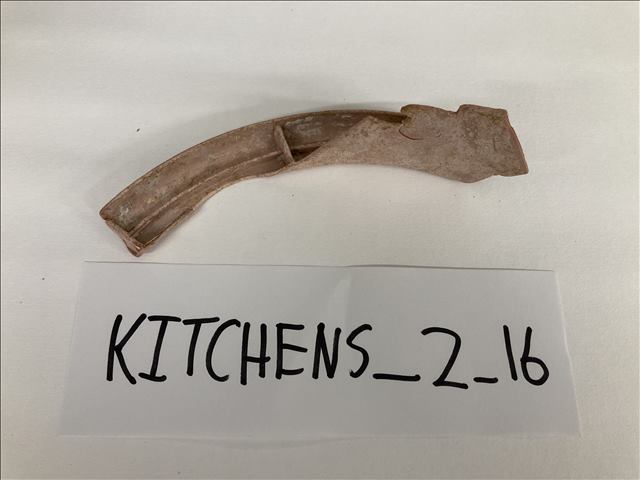 |
| PA | 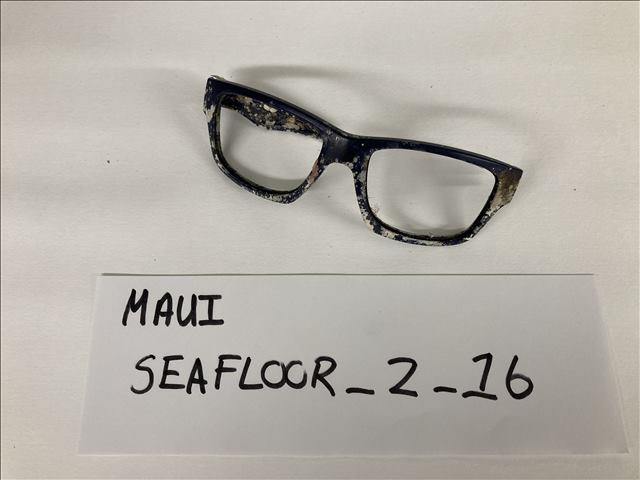 | 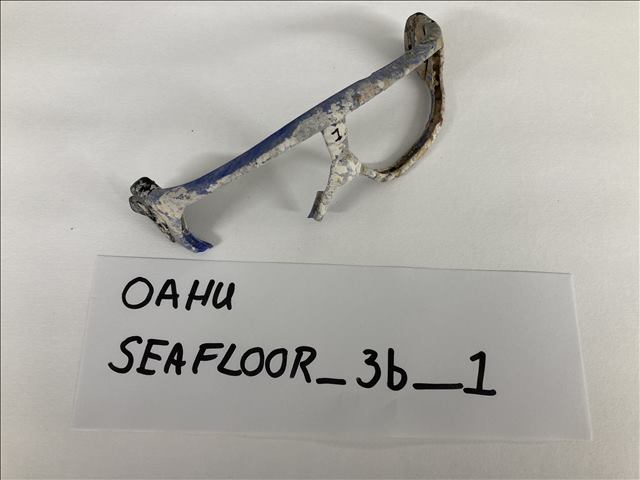 |
| PS | 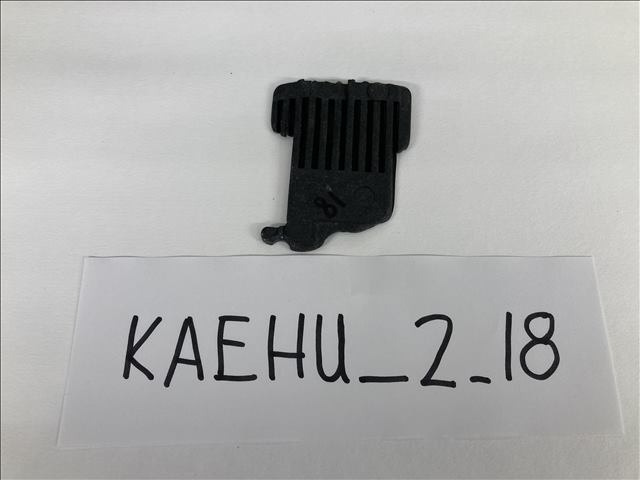 | N/A |
| **Supplemental Figure 2.** Photo images of weather plastics obtained from the Pacific Ocean prior to cryomilling and characterized as described by Brignac et al 2019. | | |

**Appendix A**

**Household Dust and Air Sampling Questionnaire**

1. Participant ID:
2. Location (street name and city):
3. Room where sampling took place?
4. Date and Time dust sampling started?
5. Date and Time dust sampling ended?
6. Date and Time air sampling started?
7. Date and Time air sampling ended?
8. Time from Gilian pump.
9. Volume from Gilian pump.
10. Housing?

Single family – 1 or 2 story  Semi-detached/joined house  Detached/free standing  Apartment  Unit/flat  Duplex  Other:

1. Year home was built?
2. Flooring in the room sampling took place?

Carpet  Area rugs  Linoleum  Wood  Laminate  Other:

1. Other type of flooring in home?

Carpet  Area rugs  Linoleum  Wood  Laminate  Other:

1. Basement:

None  Finished  Unfinished

1. Attic:

None  Finished  Unfinished

1. Number of people living in household?
2. Of those occupants, how many are younger than 5 years of age?
3. Of those occupants, how many work from home:

Full time?       Part time?       Never?

1. Is smoking allowed in the household?  Yes  No
2. Are candles/incense/burning oil used often in the household?
3. Number of pets living in household?
4. Number of times home was swept or vacuumed during the 7-day sampling period?
5. Number of times home was mopped during the 7-day sampling period?
6. Number of times home was dusted during the 7-day sampling period?
7. Primary source of heating?

Electric  Gas  Wood  Other?

1. Type of heating?

Baseboard  Radiator  Forced Air  Other?

1. Central vac?  Yes  No
2. Were any humidifiers or HEPA filters used during the 7-day sampling period?
3. Were any windows opened in the room where sampling took place?  Yes  No
4. Additional notes or comments you would like to share (i.e. other activities during sampling collection, etc)?
