# Supplementary material for: Assessment of household settled dust via silicon nanomembrane analysis pipeline (SNAP)
Source: Environ Technol Innov. Author manuscript; Available in PMC 2025 Jun 26. (PMC12201965; doi:10.1016/j.eti.2025.104106)
Supplement: MMC3 [file NIHMS2077742-supplement-MMC3.docx]

**Supplemental Figure 3. Material assessment of reference particles.** Reference particles were captured onto silicon nanomembranes, counter stained with Nile Red and Trypan Blue and imaged fluorescently. A pure plastic polymer, HDPE, a plastic polymer fiber, PET, a cellulosic fiber, Cotton, a proteinaceous fiber blend of Wool/Silk, and a weathered plastic polymer, HDPE mildly weathered. Overlay: overlaid fluorescent images of Nile Red and Trypan Blue stained particles. Greyscale: greyscale image. Nile Red: false colored fluorescence for positive Nile Red stain. Trypan Blue: false colored fluorescence for positive Trypan Blue stain. Materials characterized as plastic polymer (red), cellulosic (blue), and proteinaceous/inorganic (unstained).

**Supplemental Figure 4. Nile Red and Trypan Blue staining does not affect particle size. A**) PET and Rayon reference fibers were captured onto silicon nanomembranes, counter stained with Nile Red and Trypan Blue and imaged fluorescently. **B**) The length and width of n = 4 randomly selected reference fibers were measured using Image J and the average and standard deviations were calculated.

**Supplemental Figure 5. Surface morphology of environmental microplastics.** SEM images were taken of **A)** the oxidized outer surface and **B)** non-oxidized interior surface of a blue PE MP obtained from the Pacific Ocean’s Great Pacific Garbage Patch. This MP was cut in half to expose the non-oxidized interior surface. Polymer type was verified via Raman Spectroscopy. Red arrows indicate pore/fissure formation.
