# Supplementary figures and images for: Assessment of household settled dust via silicon nanomembrane analysis pipeline (SNAP)
Source: Environ Technol Innov. Author manuscript; Available in PMC 2025 Jun 26. (PMC12201965; doi:10.1016/j.eti.2025.104106)

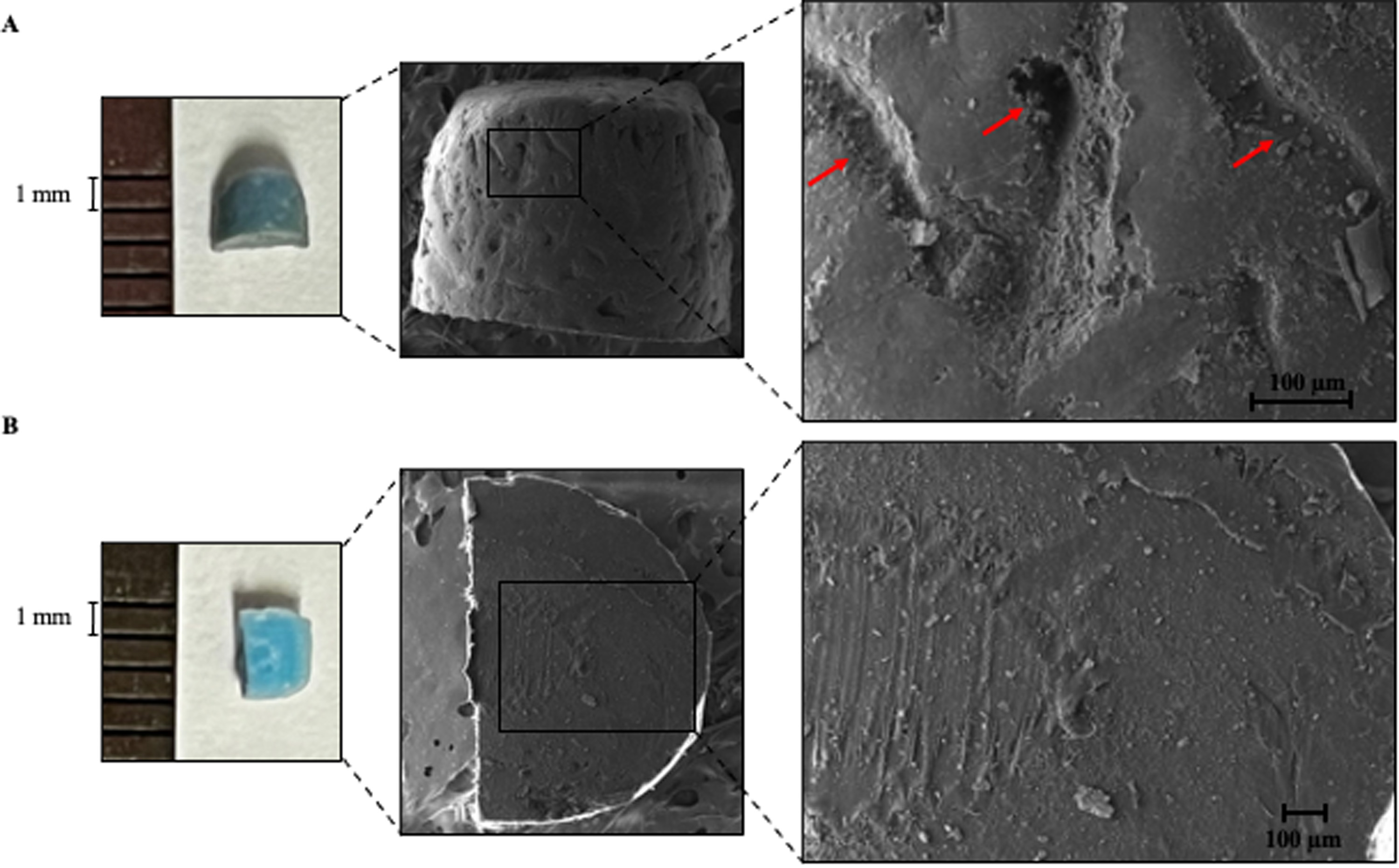

Supplement: MMC5 [file NIHMS2077742-supplement-MMC5.jpg]

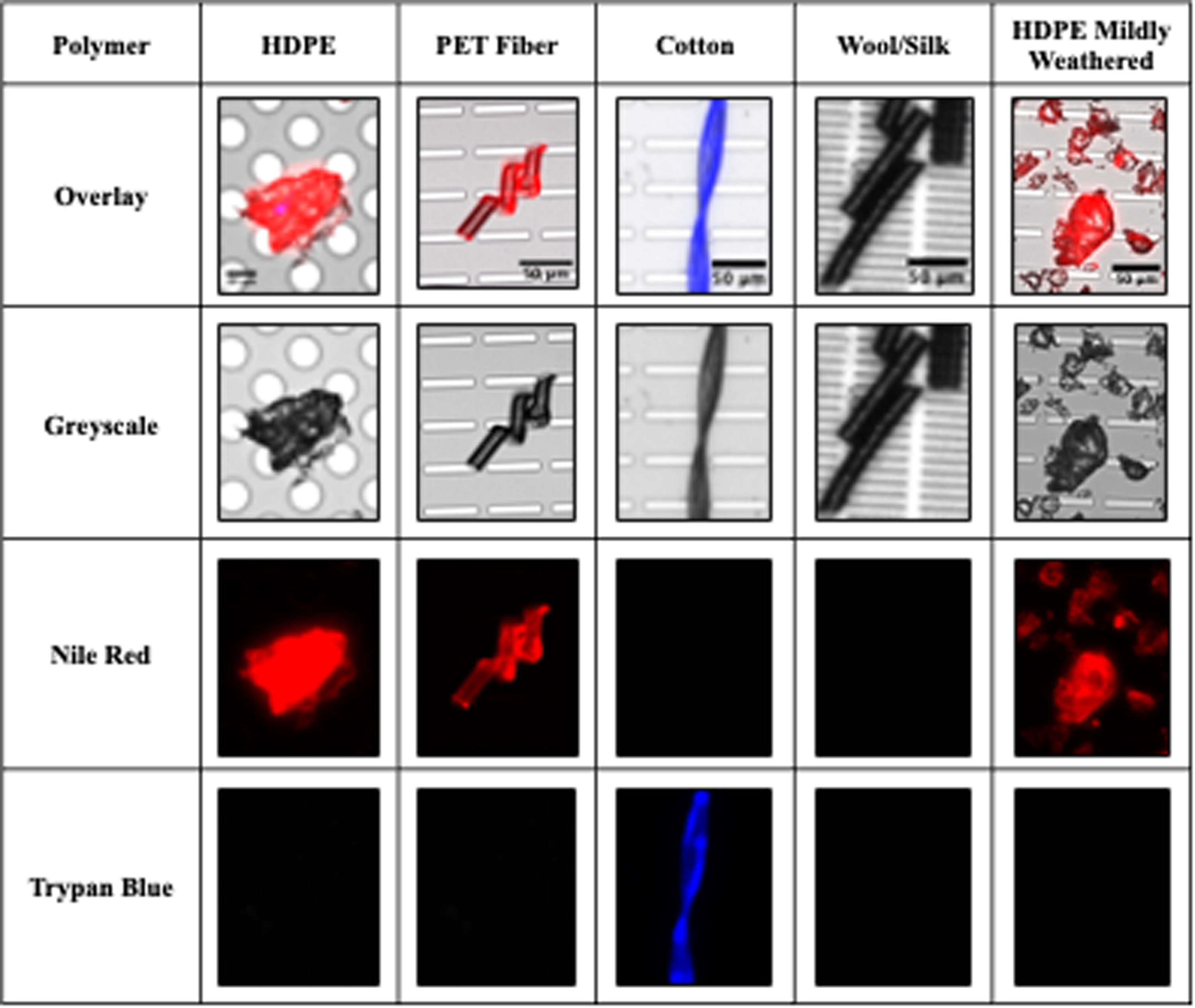

Supplement: MMC4 [file NIHMS2077742-supplement-MMC4.jpg]

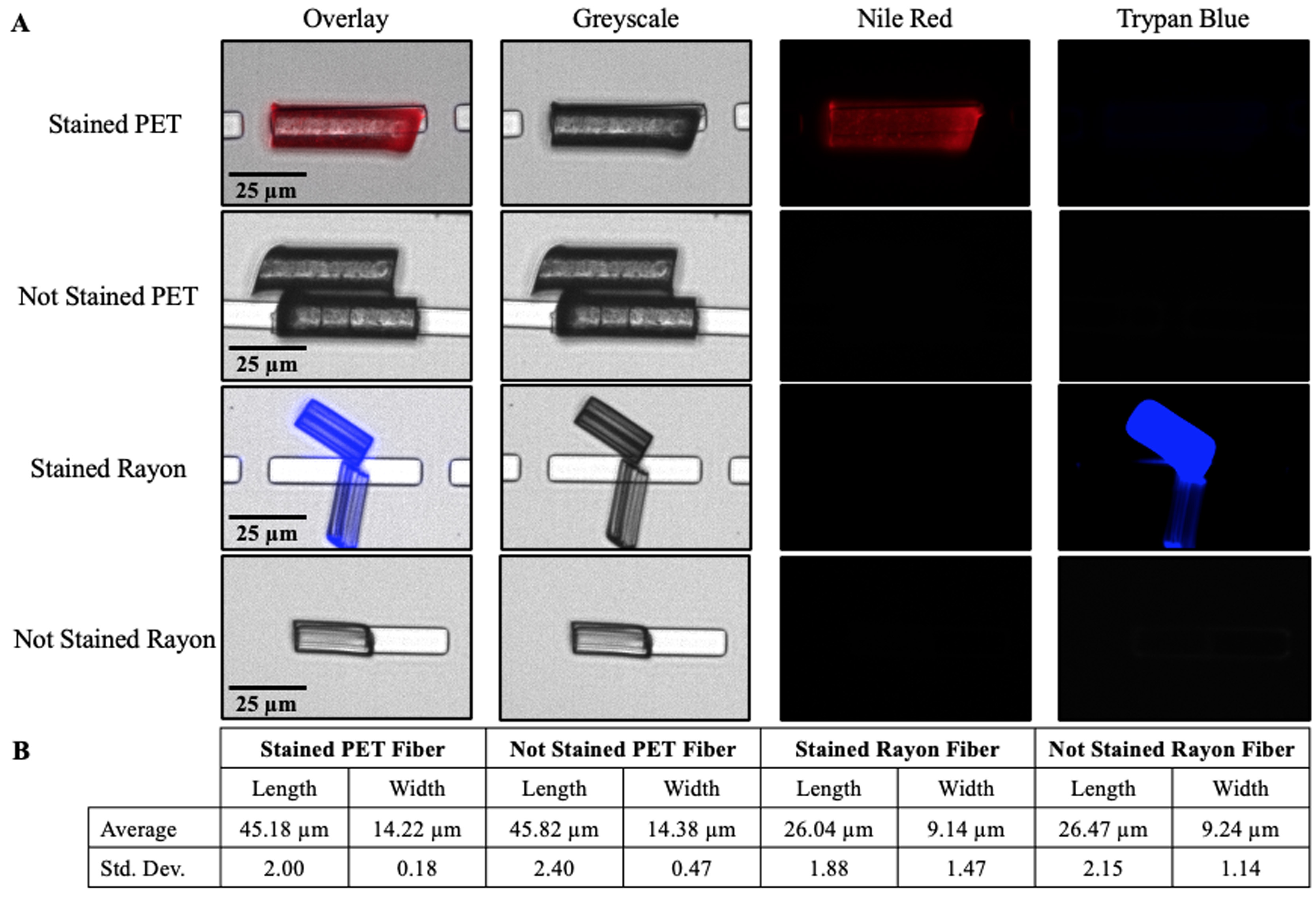

Supplement: MMC6 [file NIHMS2077742-supplement-MMC6.jpg]
